# Melanogenesis-Inducing Effect of Cirsimaritin through Increases in Microphthalmia-Associated Transcription Factor and Tyrosinase Expression

**DOI:** 10.3390/ijms16048772

**Published:** 2015-04-20

**Authors:** Hyo Jung Kim, Il Soon Kim, Yin Dong, Ik-Soo Lee, Jin Sook Kim, Jong-Sang Kim, Je-Tae Woo, Byung-Yoon Cha

**Affiliations:** 1 Research Institute for Biological Functions, Chubu University, 1200 Matsumoto, Kasugai, Aichi 487-8501, Japan; E-Mails: indersee@gmail.com (H.J.K.); dongyinsoul@gmail.com (Y.D.); jwoo@erina.co.jp (J.-T.W.); 2 KM Convergence Research Division, Korea Institute of Oriental Medicine, Daejeon 305-811, Korea; E-Mails: kisfriend@kiom.re.kr (I.S.K.); knifer48@kiom.re.kr (I.-S.L.); jskim@kiom.re.kr (J.S.K.); 3 School of Food Science and Biotechnology, Kyungpook National University, Daegu 702-701, Korea; E-Mail: vision@knu.ac.kr; 4 Department of Research and Development, Erina Co., Inc., 1-9-2 Hagashi-Shinbashi, Minato-ku, Tokyo 105-0021, Japan

**Keywords:** cirsimaritin, melanogenesis, melanoma, tyrosinase, MITF

## Abstract

The melanin-inducing properties of cirsimaritin were investigated in murine B16F10 cells. Cirsimaritin is an active flavone with methoxy groups, which is isolated from the branches of *Lithocarpus dealbatus*. Tyrosinase activity and melanin content in murine B16F10 melanoma cells were increased by cirsimaritin in a dose-dependent manner. Western blot analysis revealed that tyrosinase, tyrosinase-related protein (TRP) 1, TRP2 protein levels were enhanced after treatment with cirsimaritin for 48 h. Cirsimaritin also upregulated the expression of microphthalmia-associated transcription factor (MITF) after 24 h of treatment. Furthermore, cirsimaritin induced phosphorylation of cyclic adenosine monophosphate (cAMP) response element-binding protein (CREB) in a dose-dependent manner after treatment for 15 min. The cirsimaritin-mediated increase of tyrosinase activity was significantly attenuated by H89, a cAMP-dependent protein kinase A inhibitor. These findings indicate that cirsimaritin stimulates melanogenesis in B16F10 cells by activation of CREB as well as upregulation of MITF and tyrosinase expression, which was activated by cAMP signaling. Finally, the melanogenic effect of cirsimaritin was confirmed in human epidermal melanocytes. These results support the putative application of cirsimaritin in ultraviolet photoprotection and hair coloration treatments.

## 1. Introduction

Production of melanin in skin, hair, and eyes is required for photoprotection against ultraviolet (UV) irradiation, embryonic development, detoxification, and cosmetic coloration [1]. Melanins are synthesized in melanosomes of melanocytes through the activity of various enzymes including tyrosinase, tyrosinase-related protein (TRP) 1 and TRP2 (also known as dopachrome tautomerase). Tyrosinase catalyzes the o-hydroxylation of tyrosine (monophenol) to 3,4-dihydroxyphenylalanine (l-DOPA; diphenol) and the subsequent oxidation of DOPA to dopaquinone. Dopaquinone undergoes autocatalysis to form dopachrome. TRP1 oxidizes 5,6-dihydroxyindole-2-carboxylic acid to indole-5,6-quinone-2-carboxylic acid in mice, but not in humans. TRP2 isomerizes dopachrome to 5,6-dihydroxyindole-2-carboxylic acid [2]. Elevated levels of intracellular cyclic adenosine monophosphate (cAMP) allow activation of protein kinase A (PKA) that phosphorylates cAMP responsive element-binding protein (CREB) and CREB-binding protein, leading to an increase in the expression of microphthalmia-associated transcription factor (MITF). A study on a dominant negative mutant of MITF lacking the transactivation domain demonstrated that MITF is required for cAMP-induced stimulation of tyrosinase expression. MITF activates melanogenic gene promoters, thereby increasing melanogenesis [3,4].

The natural flavonoid cirsimaritin exhibits potent antioxidant [5,6], antibacterial [7], bacterial drug resistance [8], antispasmodic [9], and cyclooxygenase-1 inhibitory activities [10]. In addition, cirsimaritin has been reported to exhibit anticancer effects in the human gallbladder carcinoma GBC-SD cell line and a renal protective action in kidney tubular epithelial LLC-PK1 cells [11,12]. Various studies have examined the biological functions of cirsimaritin. However, its role in melanin production has not yet been investigated.

Recently, many researchers have focused on hyperpigmentation because of the beneficial effects on the improvements of disorders caused by hypopigmentation of the skin and hair. Many compounds obtained from natural sources have been demonstrated to be natural skin pigmenting products that involve in the melanogenic pathway. Several flavonoids from natural plant sources, including rhamnectin [13], tangeretin, sinensetin [14], and naringenin [15], have melanogenic effects by acting as a cofactor/substrate of tyrosinase or inducing the expression of tyrosinase.

The mitogen-activated protein kinase (MAPK) family, including ERK and p38 MAPKs, has also been implicated in mammalian melanogenesis. The involvement of ERK proteins in melanin biogenesis appears to be conserved in mammals and invertebrates. An ERK-binding domain was found to exist in hemocyanin, a latent enzyme that has tyrosinase and catecholoxidase activities in invertebrates [2]. Activation of p38 MAPK and ERK signaling induces MITF expression and tyrosinase transcription, which leads to melanin synthesis [16,17,18,19]. Thus, the activation of p38 MAPK or ERK signaling cascades may be accompanied by activation of tyrosinase, resulting in the accumulation of melanin [18,20]. Inhibition of phosphatidylinositol-3-kinase (PI3K)/Akt signaling upregulates melanogenesis by MITF phosphorylation and subsequent tyrosinase expression [20,21,22,23,24].

In the present study, we demonstrated the melanogenic activity of cirsimaritin and its molecular mechanisms in murine B16F10 melanoma cells.

## 2. Results

### 2.1. Morphological Changes Induced by Cirsimaritin

**Figure 1:**
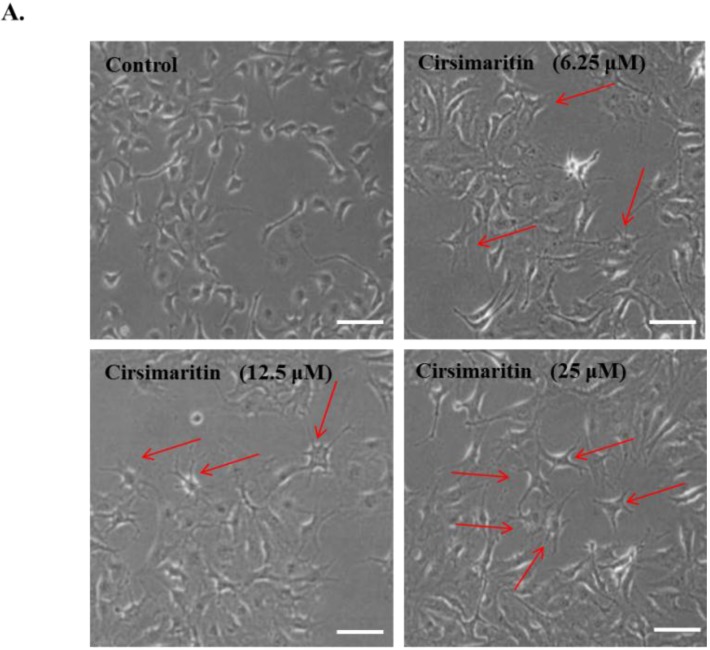

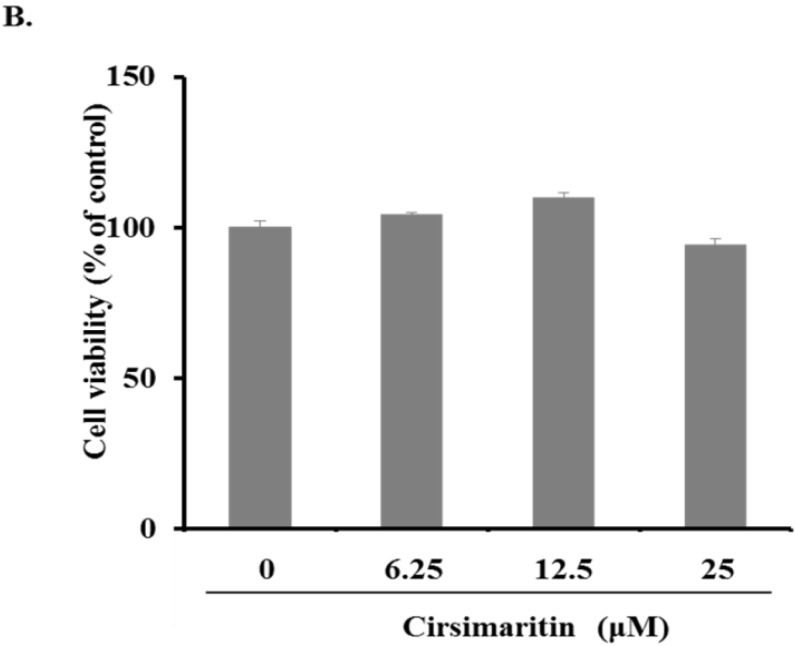
Effects of cirsimaritin on dendritogenesis and cell viability of murine B16F10 cells. Cells were treated with 0.1% (*v*/*v*) dimethylsulfoxide (DMSO) or various doses of cirsimaritin for 24 h. Morphological changes were observed under a microscope (**A**); Arrows indicate the cells having abundantly branched dendrites. Scale bar = 50 µm. Cell viability was measured by MTT assays (**B**). Results are expressed as the means ± SD of three independent experiments.

Pigmentation in melanocytes is related to an increase in melanocyte proliferation as well as stimulation of melanin neosynthesis and melanocyte dendricity, a pivotal morphological change required for melanin transfer to keratinocytes, resulting in the hyperpigmentation response of skin [25]. To examine the effect of cirsimaritin on cellular morphological changes during melanogenesis, murine B16F10 melanoma cells were treated with cirsimaritin at various concentrations (6, 12, and 25 µM) for 24 h. As a result, we observed abundantly branched dendrites in cells exposed to cirsimaritin (Figure 1A), indicating the early stages of melanogenesis. No effects on cell proliferation or cytotoxicity were observed at all tested concentrations of cirsimaritin (Figure 1B).

### 2.2. Cirsimaritin Upregulates Tyrosinase Activity and Increases Melanin Content

**Figure 2:**
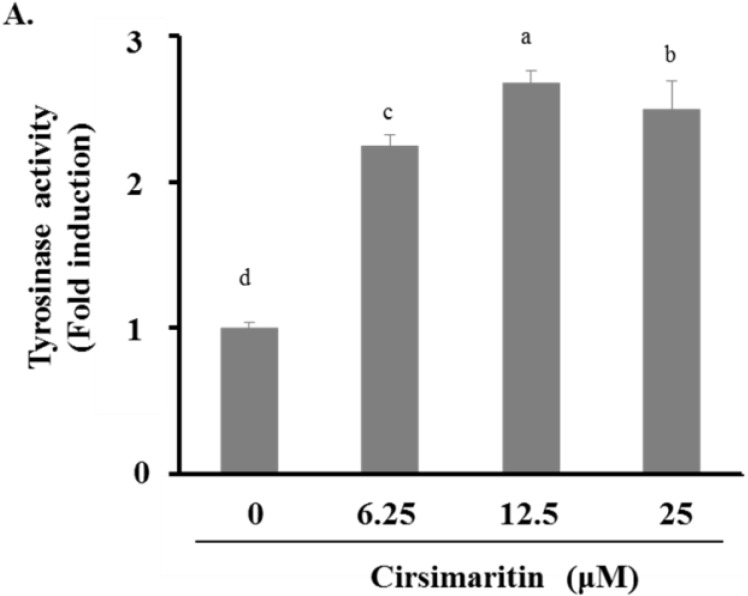

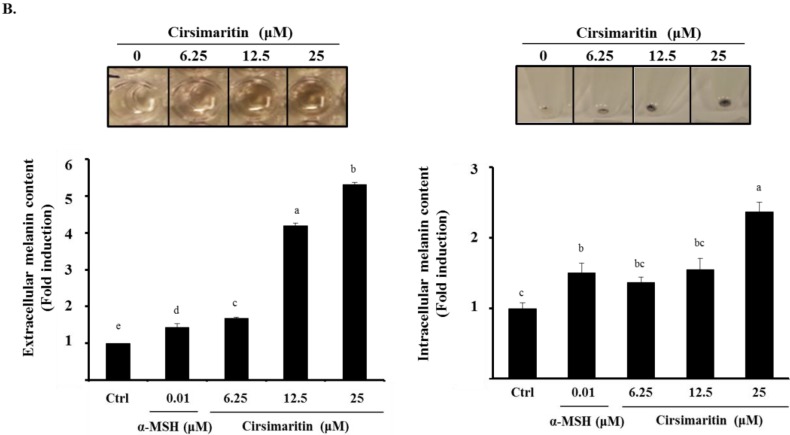
Effects of cirsimaritin on melanogenesis in B16F10 cells. Cells were treated with 0.1% (*v*/*v*) DMSO, α-MSH (0.01 μM), or cirsimaritin (6.25, 12.5, or 25 μM) for 24 (**A**) or 48 h (**B**); Images show the recovered medium and cell pellet (**B**). Tyrosinase activity and melanin content were measured as described in the Materials and Methods. Results are expressed as the means ± SD of three independent experiments. Means without a common letter differ, *p* < 0.05.

Next, we examined melanin content and tyrosine activity in cells treated with cirsimaritin. As shown in Figure 2A, tyrosinase activity was significantly increased in B16 cells treated with cirsimaritin at all examined concentrations (control, 0.46 ± 0.02; 6 μM, 1.03 ± 0.04; 12 μM, 1.22 ± 0.04; 25 μM, 1.14 ± 0.09). Cirsimaritin also caused an increase in the amounts of both intracellular and extracellular melanin in a dose-dependent manner (Figure 2B). In particular, cirsimaritin induced a 2.4-fold increase in the intracellular melanin content compared with the control group, which was higher than that induced by α-melanocyte-stimulating hormone (MSH) alone (1.5-fold increase compared with the control group).

### 2.3. Effect of Cirsimaritin on the Expression of Tyrosinase, TRP1, and TRP2

**Figure 3:**
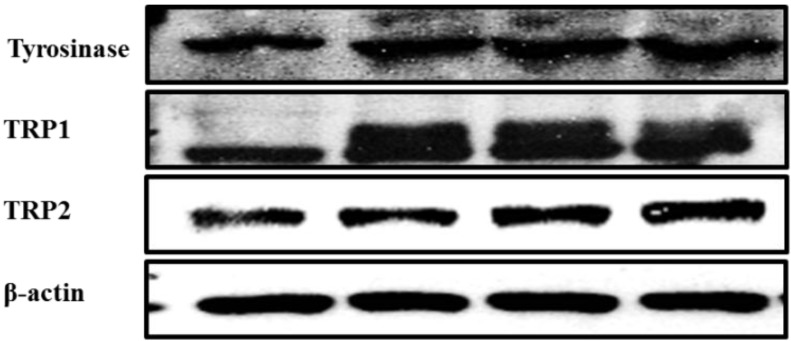

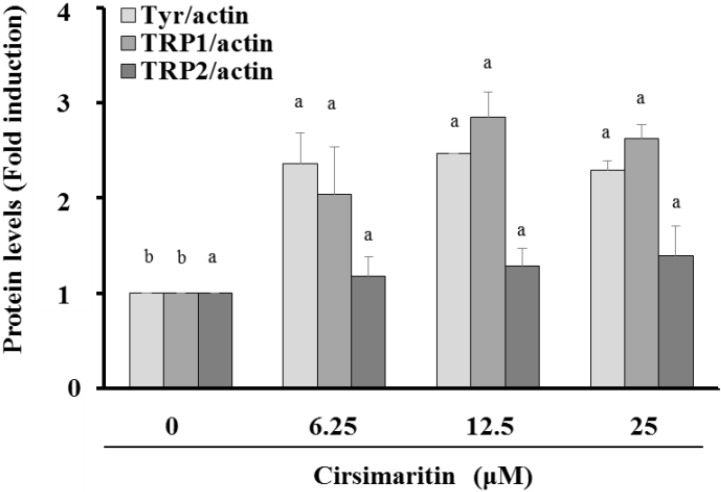
Effects of cirsimaritin on the expression of melanogenic proteins in B16F10 cells. Cells were treated with the indicated concentrations of cirsimaritin for 24 h, and then total cell lysates were subjected to western blotting for detection of tyrosinase, TRP1 and TRP2. The bar graph shows densitometric evaluation of tyrosinase, TRP1 and TRP2 relative to β-actin as the loading control. Results are expressed as the means ± SD of three independent experiments. Means without a common letter differ, *p* < 0.05.

Next, we evaluated the effects of cirsimaritin on the expression levels of tyrosinase, TRP1, and TRP2, which play important roles in melanogenesis. As shown in Figure 3, tyrosinase and TRP1 protein expression was clearly enhanced by treatment with cirsimaritin for 24 h. The optimal concentration of cirsimaritin appeared to be 5 µM because treatment with a higher concentration (25 µM) led to lower levels of tyrosinase and TRP1.

### 2.4. Effect of Cirsimaritin on Phosphorylation of CREB

**Figure 4:**
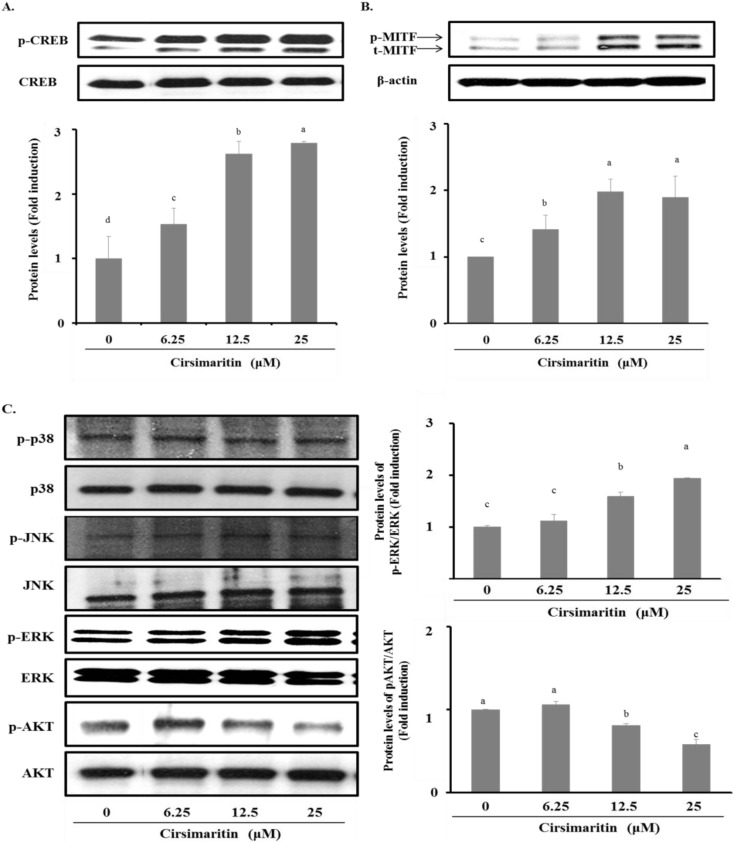
Effects of cirsimaritin on the phosphorylation of CREB and MAPKs, and the expression of MITF. Cells were treated with various concentrations of cirsimaritin for 1 h (p-CREB, p-ERK, and p-AKT) or 24 h (MITF), and then the protein expression levels were evaluated by western blotting. Bar graphs show densitometric evaluation of p-CREB (**A**); MITF (**B**); p-ERK and p-AKT (**C**) relative to CREB, β-actin, ERK, and AKT, respectively. Results are expressed as the means ± SD of three independent experiments. Means without a common letter differ, *p* < 0.05.

To clarify the mechanism of cirsimaritin in melanin synthesis, we next examined the phosphorylation of CREB, which is involved in tyrosinase activation/expression through upregulation of MITF gene expression. Western blot analysis showed dose-dependent activation of CREB in B16F10 cells treated with cirsimaritin (6, 12, or 25 μM) for 24 h (Figure 4A). Consistent with the induction of CREB phosphorylation, expression of MITF was also increased by cirsimaritin. Increases in the levels of phosphorylated MITF (p-MITF) and total MITF (t-MITF) were detected by western blotting at cirsimaritin concentrations of 12 and 25 μM (Figure 4B). These results suggest that cirsimaritin increases melanogenesis through MITF.

### 2.5. Effect of Cirsimaritin on the Activation of ERK, MAPK and AKT

**Figure 5:**
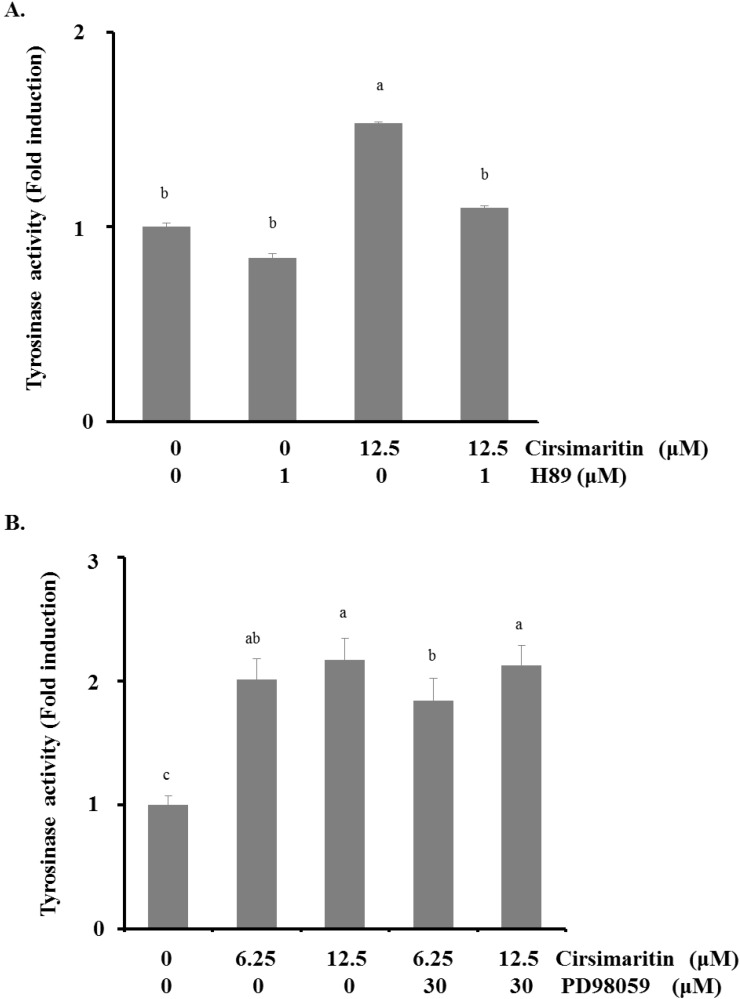
Effects of cirsimaritin with ERK and PKA inhibitor pretreatments. Cells were pretreated with H89 (1 μM) (**A**) or PD98059 (30 μM) (**B**) for 3 h before cirsimaritin treatment for 24 h, and then tyrosinase activity was measured. Results are expressed as the means ± SD of three independent experiments. Means without a common letter differ, *p* < 0.05.

To elucidate the upstream pathway of cirsimaritin in melanogenesis, we examined MAPK and AKT intracellular signal transduction cascades (Figure 4C). Phosphorylation of ERK1/2 was significantly increased after 1 h of treatment with various concentrations of cirsimaritin compared with untreated cells. Cirsimaritin did not affect the phosphorylation of p38 or JNK MAPK, but it significantly reduced the activation of AKT at 12 and 25 μM compared with the control. Next, we examined the effect of PD98059, an inhibitor of ERK, on the melanogenesis pathway in terms of tyrosinase activity to evaluate whether activation of ERK1/2 is involved in cirsimaritin-triggered melanogenesis. In contrast to our expectations, co-treatment with PD98059 did not significantly reduce the cirsimaritin-upregulated tyrosinase activity, indicating that ERK activation is not the direct pathway for melanogenesis induced by cirsimaritin (Figure 5A).

### 2.6. Melanogenesis Induced by Cirsimaritin Is Associated with the cAMP/PKA Pathway

It is well documented that cAMP accumulation induces activation of PKA, resulting in melanin synthesis. Because activation of CREB is required for cAMP responsiveness of the MITF promoter [25,26], we explored the involvement of the cAMP/PKA pathway in cirsimaritin-regulated melanogenesis. We first evaluated the effect of the PKA inhibitor H89 on cirsimaritin-induced tyrosinase activity. As shown in Figure 5B, H89 (1 μM) abrogated cirsimaritin-induced enhancement of tyrosinase activity in B16F10 cells (control, 100 ± 19.7; cirsimaritin, 153.4 ± 5.2; H89 plus cirsimaritin, 109.7 ± 12.8), implying the direct involvement of cAMP/PKA signaling in cirsimaritin-mediated melanogenesis of B16F10 cells.

### 2.7. Cirsimaritin Enhances Melanogenesis in Normal Human Epidermal Melanocytes

**Figure 6:**
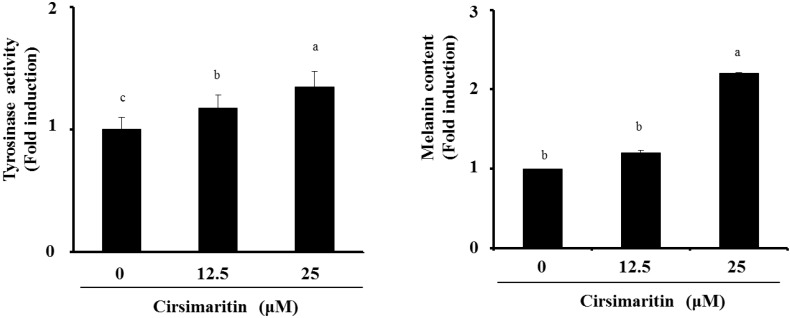
Effects of cirsimaritin on melanin content in human melanocytes. Normal human epidermal melanocytes were treated with cirsimaritin at various concentrations for 48 h, and then tyrosinase activity and melanin content were measured as described in the Materials and Methods. Results are expressed as the means ± SD of three independent experiments. Means without a common letter differ, *p* < 0.05.

To confirm the inducing effects of cirsimaritin on hyperpigmentation, we measured tyrosinase activity and melanin content in human epidermal melanocytes treated with cirsimaritin. As shown Figure 6, cirsimaritin increased not only the tyrosinase activity but also the melanin content relative to the control group in a dose-dependent manner.

## 3. Discussion

In the present study, we focused on the melanogenic activity of cirsimaritin and its molecular mechanisms, and found that cirsimaritin upregulates melanin synthesis through cAMP/PKA-dependent CREB activation and MITF upregulation in melanoma cells.

Treatment of murine melanoma cells with cirsimaritin induced clear dendritogenesis in the cell morphology, leading to melanoma cell differentiation. Tyrosinase activity and melanin content were increased in response to treatment with cirsimaritin in a dose-dependent manner. Consistent with this observation, cirsimaritin also increased tyrosinase and TRP1 protein expression levels. However, TRP2 protein expression was not clearly enhanced by cirsimaritin treatment. In melanogenesis, TRP1 affects tyrosinase activity by formation of a complex with tyrosinase and/or stabilization of tyrosinase. TRP2 controls the quantity and quality of melanin, whereas tyrosinase plays a key role in melanin biosynthesis by catalyzing the hydroxylation of tyrosine to form l-DOPA, followed by oxidation of DOPA to produce dopaquinone. Therefore, the tyrosinase-induced processes are the main step in melanin production. In the present study, although cirsimaritin did not obviously influence the gene expression of TRP2 compared with the control group, cirsimaritin treatment significantly increased tyrosinase and TRP1 protein expression, indicating that the hyperpigmentary effects of cirsimaritin are closely associated with the increase in tyrosinase expression.

Several previous studies showed that constituents extracted from plants elicit melanogenesis-inducing effects that are inhibited by the presence of a PKA inhibitor. As described in a previous study, our results also showed that cirsimaritin increased the levels of CREB phosphorylation and MITF expression. Additionally, inhibition of cAMP-dependent PKA using H89 blocked the cirsimaritin-promoted tyrosinase activity, implying that the cAMP/PKA signaling pathway is involved in cirsimaritin-mediated melanogenesis. In addition to the involvement of the cAMP/PKA/CREB/MITF pathway in melanogenesis, a possible link between MAPK and melanin synthesis has been reported previously. MAPK is suggested to be a regulator of melanocyte development. The involvement of ERK proteins in melanin biogenesis appears to be conserved in mammals and invertebrates. An ERK-binding domain was found to exist in hemocyanin, a latent enzyme that performs tyrosinase and catecholoxidase activities in invertebrates [2]. In particular, ERK-dependent phosphorylation of MITF induces melanogenesis [27]. Enhanced phosphorylation of ERK1/2 has been observed during melanogenesis in cells treated with natural compounds [14,28,29] and UV-A irradiation [30]. However, other reports indicate that activated ERK negatively regulates melanin synthesis [31,32,33]. Furthermore, treatment of melanoma cells with a specific inhibitor of the MAPK pathway or overexpression of dominant negative mutants of MEK induce melanogenesis by increasing tyrosinase expression [33]. ERK activation can also lead to attenuation of melanogenesis by accelerating MITF degradation [34]. In our study, cirsimaritin treatment led to ERK1/2 phosphorylation in a dose-dependent manner. However, treatment with the ERK inhibitor PD98059 did not result in a significant reduction of cirsimaritin-stimulated tyrosinase activity. Incomplete inhibition of the cirsimaritin-induced increase of tyrosinase activity by the ERK inhibitor implies that mechanisms other than ERK activation are involved in the melanogenic effect of cirsimaritin. In addition to MAPKs, previous studies have indicated that the PI3K/AKT signaling pathway is related to melanogenesis in B16 melanoma cells. Activation of PI3K/Akt suppresses melanin synthesis by reducing MITF expression and the activation of downstream signal transduction pathways, including tyrosinase, in α-MSH-induced hyperpigmentation [35]. Similarly, we also confirmed a decrease in Akt phosphorylation in cells treated with cirsimaritin, indicating that the PI3K/Akt pathway is related to cirsimaritin-triggered melanogenesis. Additionally, the melanogenic effect of cirsimarin was confirmed by upregulated levels of tyrosinase activity and melanin production in normal human epidermal melanocytes.

Currently, there are no commercial products for induction of hyperpigmentation. Several compounds have been researched, but they did not successfully induce hyperpigmentation in human skin. From this point of view, cirsimaritin is a superior natural compound for induction of melanogenesis and can be used in pigmentation cosmetics. However, cirsimaritin has no wide distribution in South East Asia. Therefore, despite the outstanding effects of cirsimaritin on hyperpigmentation, there is still some difficulty in purifying and obtaining sufficient amounts for commercial application.

**Scheme 1:**
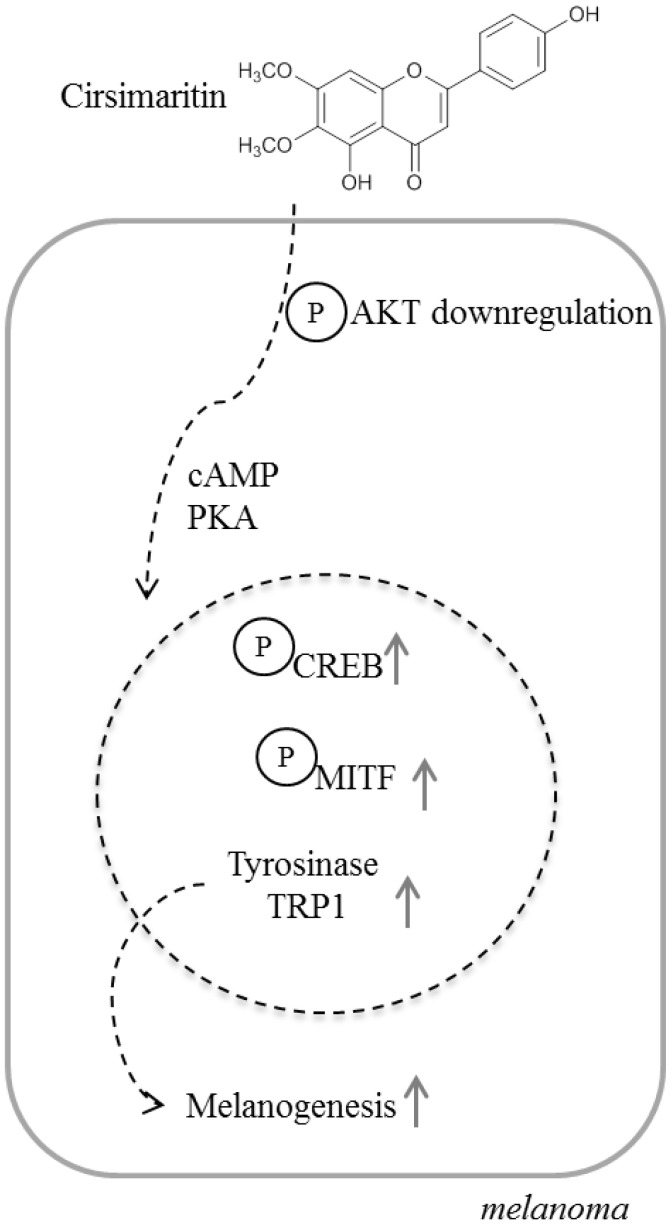
Cirsimaritin induces melanogenesis by stimulating the expression of tyrosinase and TRP1 through the cAMP/PKA cascade and subsequent activation of CREB and MITF in B16F10 cells.

In summary, the present study is the first to examine the hyperpigmentation effect of cirsimaritin and elucidate the molecular mechanism by which cirsimaritin induces melanogenesis in murine melanoma cells. Our results show that cirsimaritin stimulates the activity and expression of tyrosinase via activation of cAMP/PKA signaling and subsequent phosphorylation of CREB and upregulation of MITF, tyrosinase, and TRP1 expression (Scheme 1). These results suggest that cirsimaritin could be used as a component in sunscreen agents for photoprotection without the risk of causing skin damage or as a component of hair color treatments for grey hair. Further studies are required to assess the safety and efficacy of cirsimaritin.

## 4. Materials and Methods

### 4.1. Chemicals and Reagents

EtOH, EtOAC, BuOH, CHCl_3_, MeOH, CAN, dimthylsulfoxide (DMSO), sodium dodecyl sulfate (SDS), phenylmethanesulfonylfluoride (PMSF), and sodium deoxycholate were purchased from Wako Pure Chemical Industries Ltd. (Osaka, Japan). Triton X-100, l-DOPA, and 3-methyl-2-benzothiazolinone hydrozone hydrochloride hydrate (MBTH) were purchased from Sigma-Aldrich (St. Louis, MO, USA) and 3-(4,5-dimethyl-thiazol-2-yl)-2,5-diphenyltetrazolium bromide (MTT) was from Dojindo Laboratories (Kumamoto, Japan). Eagle’s Minimal Essential Medium (EMEM) was obtained from Wako Pure Chemical Industries Ltd. Fetal bovine serum and penicillin-streptomycin were purchased from Life Technologies (Carlsbad, CA, USA). Antibodies against tyrosinase (polyclonal anti-mouse, SC-7834), TRP1 (polyclonal anti-mouse, SC-25543) and TRP2 (polyclonal anti-mouse, SC-25544) were purchased from Santa Cruz Biotechnology (Dallas, TX, USA), Antibodies against p-CREB (polyclonal anti-mouse, 9191), CREB (monoclonal anti-mouse, 9104), p-AKT (monoclonal anti-mouse, 4060), AKT (monoclonal anti-mouse, 4691), p-p38 (monoclonal anti-mouse, 9215), p38 (polyclonal anti-mouse, 9212), p-ERK (polyclonal, anti-mouse, 4370), ERK (polyclonal anti-mouse, 9102), and β-actin (polyclonal anti-mouse, 4967) were obtained from Cell Signaling Technology (Danvers, MA, USA). The anti-MITF antibody (monoclonal anti-mouse, MAB3747-I) was obtained from Millipore (Temecula, CA, USA).

### 4.2. Isolation of Cirsimaritin

**Figure 7:**
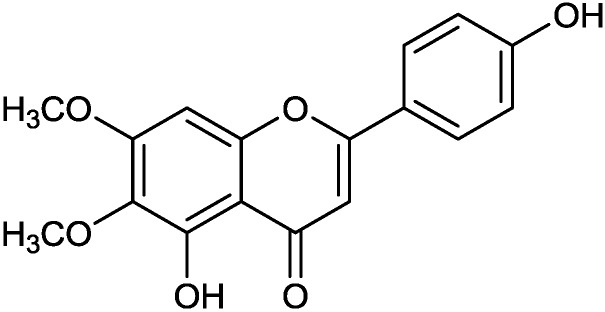
Chemical structure of cirsimaritin.

Air-dried branches of *Lithocarpus dealbatus* (6 kg) were extracted with 80% aqueous EtOH (three times, 30 L each) at room temperature for 7 days, filtered, and concentrated to generate an 80% EtOH extract (720 g). The extract (150 g) was suspended in H_2_O (4 L) and partitioned successively with *n*-hexane (3 × 4 L), EtOAc (3 × 4 L), and *n*-BuOH (3 × 4 L) to generate *n*-hexane- (11.9 g), EtOAc- (42.3 g), and *n*-BuOH-soluble fractions (25.4 g), respectively. The EtOAc-soluble fraction (41 g) was subjected to silica gel column chromatography (70–230 μm, 50 cm × 7 cm i.d.) and eluted with a gradient solvent system of CHCl_3_-MeOH (100:1 → 0:1) to generate 11 fractions (A–K). Of these fractions, fraction E (248 mg) was purified further by extensive preparative reversed-phase high performance liquid chromatography (Agilent 1200 system; Chemcobond 5-ODS-W column (5.0 μm, 250 mm × 10 mm i.d.); mobile phase, 40% ACN in water; UV detection, 254 nm; flow rate, 4.0 mL/min) to obtain cirsimaritin (28 mg, Figure 7).

### 4.3. Cell Culture

Murine melanoma B16F10 cells (CRL-6415) were obtained from the American Type Culture Collection (Manassas, VA, USA) RIKEN BioResource Center RIKEN BioResource Center. Cells were seeded in 35-mm dishes at a density of 2 × 10^5^ cells with EMEM containing 10% fetal bovine serum, 100 U/mL penicillin, and 0.l mg/mL streptomycin, and incubated at 37 °C with 5% CO_2_ for 24 h. The cells were treated with various concentrations of cirsimaritin for 24 (for tyrosinase activity assays) or 48 h (for melanin content assays). Human epidermal melanocytes (moderately pigmented donor, HEMn-MP) were obtained from Life Technologies. The cells were seeded in 6-well plates at a density of 2 × 10^5^ cells in 254 medium (Life Technologies) containing 1% human melanocyte growth supplements, 100 U/mL penicillin, and 0.l mg/mL streptomycin (Life technologies), and incubated at 37 °C with 5% CO_2_ for 24 h. Then, the medium was replaced with 254 medium containing transferrin (5 μg/mL), insulin (5 μg/mL), heparin (3 μg/mL), and hydrocortisone (0.18 μg/mL), and the cells were further incubated for 48 h. Human melanoma MMAc cells were purchased from RIKEN BioResource Center (Ibaraki, Japan) and cultured with Dulbecco’s modified Eagle’s medium (Wako Pure Chemical Industries Ltd.) containing 10% fetal bovine serum, 100 U/mL penicillin, and 0.l mg/mL streptomycin. The cells were treated with cirsimaritin for 48 h and then subjected to melanin content or tyrosinase activity assays.

### 4.4. Cell Morphology and Cell Viability Measurement

Cell morphology and pigmentation were examined under a CKX41 microscope (Olympus Optical Co., Ltd., Tokyo, Japan). Cell cultures were analyzed on day 1 by removing the medium and adding an MTT solution (0.5 mg/mL in phenol red-free culture medium). After 2 h of incubation, the solution was replaced with 200 μL DMSO. After 10 min, the absorbance was determined at 570 nm (ref. 630 nm [36]) by a microplate spectrophotometer (Dai-Nippon Pharmaceutical Co., Osaka, Japan). The cell proliferation of each group was calculated as the absorbance of the treatment group relative to the control.

### 4.5. Measurement of Cellular Tyrosinase Activity

Tyrosinase activity in B16F10 cells was assayed as described previously with modifications. Cells were washed using cold PBS and lysed with 1% sodium deoxycholate and 0.5% Triton X-100 for 2 h. Next, 150 μL of a reaction mixture containing solutions A, B, and C (2:1:1) (solution A, 2% *N*,*N*-dimethylformamide in 100 mM sodium phosphate (pH 7.1); solution B, 5 mM l-DOPA in 100 mM sodium phosphate (pH 7.1); solution C, 20 mM MBTH in H_2_O) was added to the cells, followed by incubation at 37 °C for 10 min. Oxidation of l-DOPA to dopachrome was measured at 505 nm (ref. 490 nm [37]) by the microplate spectrophotometer. Some of the lysate was applied to protein assays using a protein assay reagent kit (Thermo Scientific, Waltham, MA, USA). The protein concentration of the samples was determined by a standard curve of bovine serum albumin. The tyrosinase activity of each sample was normalized to the protein content and calculated as the relative fold induction of the treatment group to the control.

### 4.6. Melanin Content Measurement

After treatment with cirsimaritin, the extracellular melanin content in the cell culture medium (phenol red-free) was measured at 405 nm (ref. 570 nm [38]) using the microplate spectrophotometer. The cells were then washed twice with ice-cold PBS, lysed with RIPA buffer (50 mM Tris-HCl, 150 mM NaCl, 1 mM EDTA, 1% Triton X-100, 1% sodium deoxycholate, 0.1% SDS, pH 7.4, 1 mM PMSF and protease inhibitors), and centrifuged at 10,000× *g* for 20 min. Supernatants were analyzed for the protein concentration, and pellets were solubilized in 200 μL of 1 M NaOH for 2 h at 60 °C. The absorbance was determined spectrophotometrically at 405 nm (ref. 570 nm [38]) by the microplate spectrophotometer. A portion of the lysate was used for protein assays. The protein concentration of each sample was determined by a standard curve. Extracellular and intracellular melanin contents were calculated by normalizing the total melanin values to the protein content and expressed as the relative fold induction of the treatment group to the untreated group.

### 4.7. Western Blotting

Cell lysates were obtained by extraction with RIPA lysis buffer. The lysates were then vortexed for 15 s every 10 min for a total of 40 min on ice and then centrifuged (10,000× *g*, 20 min). After boiling with sample buffer, 10 µg of proteins were separated on 10% SDS polyacrylamide gels at 80–150 V and then transferred onto membranes for 60 min at 100 V. The membrane was blocked for 30 min with 5% skim milk solution and then incubated with a primary antibody at a dilution of 1:1000 at 4 °C overnight. The protein bands were detected using a chemiluminescence kit (Amersham Biosciences, Buckinghamshire, UK) according to the manufacturer’s instructions and visualized by exposure to chemiluminescence film (Fujifilm Corporation, Tokyo, Japan).

### 4.8. Statistical Analysis

Data were assessed by analysis of variance followed by Duncan’s multiple range test using SPSS software (SPSS Inc., Chicago, IL, USA). The level of significance was set at *p* < 0.05.

## References

[1] Sturm R.A. (2002). Skin colour and skin cancer-MC1R, the genetic link. Melanoma Res..

[2] Coates C.J., Nairn J. (2014). Diverse immune functions of hemocyanins. Dev. Comp. Immunol..

[3] Hearing V.J. (1999). Biochemical control of melanogenesis and melanosomal organization. J. Investig. Dermatol..

[4] Screaton R.A., Conkright M.D., Katoh Y., Best J.L., Canettieri G., Jeffries S., Guzman E., Niessen S., Yates J.R., Takemori H. (2004). The CREB coactivator TORC2 functions as a calcium- and cAMP-sensitive coincidence detector. Cell.

[5] Ben Sghaier M., Skandrani I., Nasr N., Franca M.G., Chekir-Ghedira L., Ghedira K. (2011). Flavonoids and sesquiterpenes from Tecurium ramosissimum promote antiproliferation of human cancer cells and enhance antioxidant activity: A structure-activity relationship study. Environ. Toxicol. Pharmacol..

[6] Nyiligira E., Viljoen A.M., van Heerden F.R., van Zyl R.L., van Vuuren S.F., Steenkamp P.A. (2008). Phytochemistry and *in vitro* pharmacological activities of South African Vitex (Verbenaceae) species. J. Ethnopharmacol..

[7] Isobe T., Doe M., Morimoto Y., Nagata K., Ohsaki A. (2006). The anti-Helicobacter pylori flavones in a Brazilian plant, *Hyptis fasciculata*, and the activity of methoxyflavones. Biol. Pharm. Bull..

[8] Maia G.L., Falcão-Silva Vdos S., Aquino P.G., de Araújo-Júnior J.X., Tavares J.F., da Silva M.S., Rodrigues L.C., de Siqueira-Júnior J.P., Barbosa-Filho J.M. (2011). Flavonoids from *Praxelis clematidea* R.M. King and Robinson modulate bacterial drug resistance. Molecules.

[9] Weimann C., Goransson U., Pongprayoon-Claeson U., Claeson P., Bohlin L., Rimpler H., Heinrich M. (2002). Spasmolytic effects of *Baccharis conferta* and some of its constituents. J. Pharm. Pharmacol..

[10] Kelm M.A., Nair M.G., Strasburg G.M., DeWitt D.L. (2000). Antioxidant and cyclooxygenase inhibitory phenolic compounds from *Ocimum sanctum* Linn. Phytomedicine.

[11] Quan Z., Gu J., Dong P., Lu J., Wu X., Wu W., Fei X., Li S., Wang Y., Wang J. (2010). Reactive oxygen species-mediated endoplasmic reticulum stress and mitochondrial dysfunction contribute to cirsimaritin-induced apoptosis in human gallbladder carcinoma GBC-SD cells. Cancer Lett..

[12] Yokozawa T., Dong E., Kawai Y., Gemba M., Shimizu M. (1999). Protective effects of some flavonoids on the renal cellular membrane. Exp. Toxicol. Pathol..

[13] Kubo I., Kinst-Hori I. (1999). Flavonols from saffron flower: Tyrosinase inhibitory activity and inhibition mechanism. J. Agric. Food Chem..

[14] Yoon H.S., Lee S.R., Ko H.C., Choi S.Y., Park J.G., Kim J.K., Kim S.J. (2007). Involvement of extracellular signal-regulated kinase in nobiletin-induced melanogenesis in murine B16/F10 melanoma cells. Biosci. Biotechnol. Biochem..

[15] Ohguchi K., Akao Y., Nozawa Y. (2006). Stimulation of melanogenesis by the citrus flavonoid naringenin in mouse B16 melanoma cells. Biosci. Biotechnol. Biochem..

[16] Ahn J.H., Jin S.H., Kang H.Y. (2008). LPS induces melanogenesis through p38 MAPK activation in human melanocytes. Arch. Dermatol. Res..

[17] Widlund H.R., Fisher D.E. (2003). Microphthalmia-associated transcription factor: A critical regulator of pigment cell development and survival. Oncogene.

[18] Hirata N., Naruto S., Ohguchi K., Akao Y., Nozawa Y., Iinuma M., Matsuda H. (2007). Mechanism of the melanogenesis stimulation activity of (−)-cubebin in murine B16 melanoma cells. Bioorg. Med. Chem..

[19] Kim D.S., Hwang E.S., Lee J.E., Kim S.Y., Kwon S.B., Park K.C. (2003). Sphingosine-1-phosphate decreases melanin synthesis via sustained ERK activation and subsequent MITF degradation. J. Cell. Sci..

[20] Jiang Z., Xu J., Long M., Tu Z., Yang G., He G. (2009). 2,3,5,40-Tetrahydroxystilbene-2-*O*-β-d-glucoside (THSG) induces melanogenesis in B16 cells by MAP kinase activation and tyrosinase upregulation. Life Sci..

[21] Khaled M., Larribere L., Bille K., Aberdam E., Ortonne J.P., Ballotti R., Bertolotto C. (2002). Glycogen synthase kinase 3 is activated by cAMP and plays an active role in the regulation of melanogenesis. J. Biol. Chem..

[22] Khaled M., Larribere L., Bille K., Ortonne J.P., Ballotti R., Bertolotto C. (2003). Microphthalmia associated transcription factor is a target of the phosphatidylinositol-3-kinase pathway. J. Investig. Dermatol..

[23] Lee J., Jung K., Kim Y.S., Park D. (2007). Diosgenin inhibits melanogenesis through the activation of phosphatidylinositol-3-kinase pathway (PI3K) signaling. Life Sci..

[24] Hirose N., Inoue T., Nishihara K., Sugano M., Akimoto K., Shimizu S., Yamada H. (1991). Inhibition of cholesterol absorption and synthesis in rats by sesamin. J. Lipid Res..

[25] Buscà R., Ballotti R. (2000). Cyclic AMP a key messenger in the regulation of skin pigmentation. Pigment Cell Res..

[26] Price E.R., Ding H.F., Badalian T., Bhattacharya S., Takemoto C., Yao T.P. (1998). Lineage-specific signaling in melanocytes. C-kit stimulation recruits p300: CBP to microphthalmia. J. Biol. Chem..

[27] Hemesath T.J., Price E.R., Takemoto C., Badalian T., Fisher D.E. (1998). MAPK links microphthalmia to c-kit signaling in melanocytes. Nature.

[28] Horibe I., Satoh Y., Shiota Y., Kumagai A., Horike N., Takemori H., Uesato S., Sugie S., Obata K., Kawahara H. (2013). Induction of melanogenesis by 4'-*O*-methylated flavonoids in B16F10 melanoma cells. J. Nat. Med..

[29] Lee J., Jung E., Park J., Jung K., Park E., Kim J., Hong S., Park J., Park S., Lee S. (2005). Glycyrrhizin induces melanogenesis by elevating a cAMP level in B16 melanoma cells. J. Investig. Dermatol..

[30] Yanase H., Ando H., Horikawa M., Watanabe M., Mori T., Matsuda N. (2001). Possible involvement of ERK1/2 in UVA-induced melanogenesis in cultured normal human epidermal melanocytes. Pigment Cell Res..

[31] Wu M., Hemesath T.J., Takemoto C.M., Horstmann M.A., Wells A.G., Price E.R., Fisher D.Z., Fisher D.E. (2000). C-kit triggers dual phosphorylations, which couple activation and degradation of the essential melanocyte factor Mi. Genes Dev..

[32] Kim D.S., Kim S.Y., Lee J.E., Kwon S.B., Joo Y.H., Youn S.W., Park K.C. (2003). Sphingosine-1-phosphate-induced ERK activation protects human melanocytes from UVB-induced apoptosis. Arch. Pharm. Res..

[33] Englaro W, Bertolotto C., Buscà R., Brunet A., Pagès G., Ortonne J.P., Ballotti R. (1998). Inhibition of the mitogen-activated protein kinase pathway triggers B16 melanoma cell differentiation. J. Biol. Chem..

[34] Lee C.S., Park M., Han J., Lee J.H., Bae I.H., Choi H., Son E.D., Park Y.H., Lim K.M. (2013). Liver X receptor activation inhibits melanogenesis through the acceleration of ERK-mediated MITF degradation. J. Investig. Dermatol..

[35] Jang J.Y., Lee J.H., Jeong S.Y., Chung K.T., Choi Y.H., Choi B.T. (2009). Partially purified *Curcuma longa* inhibits α melanocyte stimulating hormone stimulated melanogenesis through extracellular signal regulated kinase or Akt activation mediated signalling in B16F10 cells. Exp. Dermatol..

[36] Kim H.J., Jung C.L., Jeong Y.S., Kim J.S. (2014). Soybean-derived glyceollins induce apoptosis through ROS generation. Food Funct..

[37] Winder A.J., Harris H. (1991). New assays for the tyrosine hydroxylase and dopa oxidase activities of tyrosinase. Eur. J. Biochem..

[38] Cho M., Ryu M., Jeong Y., Chung Y.H., Kim D.E., Cho H.S., Kang S., Han J.S., Chang M.Y., Lee C.K. (2009). Cardamonin suppresses melanogenesis by inhibition of Wnt/β-catenin signaling. Biochem. Biophys. Res. Commun..

